# Stiffness of the locking compression plate as an external fixator for treating distal tibial fractures: a biomechanics study

**DOI:** 10.1186/s12891-016-1384-1

**Published:** 2017-01-19

**Authors:** Wei Liu, Lihui Yang, Xiaochuan Kong, Likun An, Gang Hong, Zicheng Guo, Lei Zang

**Affiliations:** Department of Orthopedics, Beijing Chao Yang Hospital, Capital Medical University, Beijing, 100020 China

**Keywords:** External fixation, Tibia, Fracture, Locking compression plate, Biomechanics

## Abstract

**Background:**

Locking compress plate, as external fixator, is an attractive technique for distal tibial fracture treatment. But it still remains unclear whether the external LCP has sufficient stiffness. Thus, the present study aims to make a comprehensive evaluation of the stiffness of external locking compress plate when it is used as an external fixator in distal tibial fractures treatment.

**Methods:**

Composite tibia was used to simulate distal tibia fracture (Orthopedic Trauma Association type 43 A3 fracture). The fractures were stabilized with medial distal tibial locking compress plates (LCP group), medial distal tibial locking compress plates with 30-mm plate-bone distances (EF-tibia group), and medial distal femur locking compress plates with 30-mm plate-bone distances (EF-femur group). Stiffness of each configuration was measured under axial compression loading and in axial torsion loading directions. Compression stiffness and torsional rigidity were compared across different groups.

**Results:**

Compared with LCP group, (1) EF-tibia group showed significantly lower (*p* < 0.001) compression stiffness and torsional rigidity; (2) EF-femur group showed significantly lower (*p* < 0.001) compression stiffness, but significantly higher (*p* < 0.001) torsional rigidity.

**Conclusions:**

The results indicated that locking compress plate as an external fixator was flexible, and the distal femur locking compress plate was preferred over the distal tibial locking compress plate to be an external fixator in distal tibia fracture treatment.

## Background

Distal tibial fracture is a common type of bone fractures, but its treatment is still a challenge for orthopedic surgeons in clinical practice. Neither traditional treatment methods like non-operative managements as well as open reduction and inner fixation (e.g., intramedullary nailing (IM), plate fixation and external fixation (EF)) nor minimally invasive percutaneous plate osteosynthesis (MIPPO) which was recently proposed can achieve satisfied treatment effects. Non-operative managements can avoid infections as well as implant-related complications that often appear after operations, and are popular in patients who are intolerantto anesthesia; however, this kind of treatments often companies with tibial shortening and angular malunion [1]. IM is associated with a high rate of union and a reduction of disturbance to the soft-tissue envelope as well as blood supplies at the fracture site; nevertheless, good reduction is very difficult to be acquired and maintained with IM strategy [2]. EF is a preferred strategy in the first stage treatment of the two-stage protocol for severe high-energy tibial fractures, but is usually with complications like ankle stiffness, pin site infection, and pin loosening [3]. Particularly, the rates of infection are kept relatively high in the open reduction and internal fixation [4]. MIPPO was developed to reduce irritation and damage to soft tissue, and was reported to do well in alignment, union, as well as holding low infection rates in some basic and clinical studies [5]; however, sample sizes in these studies were quite small, making it difficult to draw consolidated conclusions. A study has reported that MIPPO might lead to relative high rates of complications including disturbances of fracture healing, wound complications and axial malalignment [6], indicating that risks still exist for this newly developed treatment method. In addition, there was even a systematic review showing contradictory results for MIPPO advantages, i.e., the complication rates of MIPPO treatment protocol were not significantly different from those in traditional open techniques [7], further challenging the efficacy of MIPPO.

Locking compress plate (LCP), which is lightweight, comfortable and convenient for patients to ambulate [8], is very attractive to be used as external fixators in the treatment of distal tibial fracture, especially compared with conventional external fixators. LCP has been successfully used in open or closed distal tibial fractures and shown good rates of union and ankle-joint motion [8–11] due to the use of angular stable anchoring of screws in the plate, which enables LCP to stabilize the short distal tibial segment without spanning the ankle joint. However, the stability of LCP as an external fixator was questioned by several studies [12–14] based on the fact that stiffness of compression and torsion of plate would be significantly reduced when the distance between the plate and the bone surface was above 5 mm [12]. A poor stability of external fixator may cause excessive interfragmentary movements during weight-bearing functional exercises, prolonging the healing period and causing delayed union or nonunion of the bone fragments.

Thus, the present study aims to quantitatively evaluate the stiffness of compression and torsion of external LCP fixator in the treatment of distal tibial fractures. The stiffness of different tibial configurations (LCP, EF-femur, and EF-tibia) was measured under axial compression loading and in axial torsion loading directions. Compression stiffness and torsional rigidity were then comprehensively compared across different configurations.

## Methods

### Specimen preparation

Thirty-six composite tibiae (Sawbones #3401, Pacific Research Laboratories, Inc., Vashon, WA, USA) were used in the biomechanics test. An osteotomy was performed in the transverse section at 40 mm and 50 mm above the tibial plafond, with a 10-mm section removed to simulate an unstable distal tibia fracture (Orthopedic Trauma Association type 43 A3 fracture). To examine the influences of bone-plate distance as well as diameters of the screws and dimensions of the plate, three different configurations, i.e., LCP as external fixations (LCP group), medial distal tibial LCP with 30-mm plate-bone distances (EF-tibia group), and medial distal femur LCP with 30-mm plate-bone distances (EF-femur group), were used to stabilize the fractures. In the third group, medial distal femur LCP was used instead of LCP Metaphyseal Plate. That is because medial distal femur LCP is more rigid due to its broad end as well as the allowance of a maximum number of locking screws to stabilize the short distal segment.

**Fig. 1:**
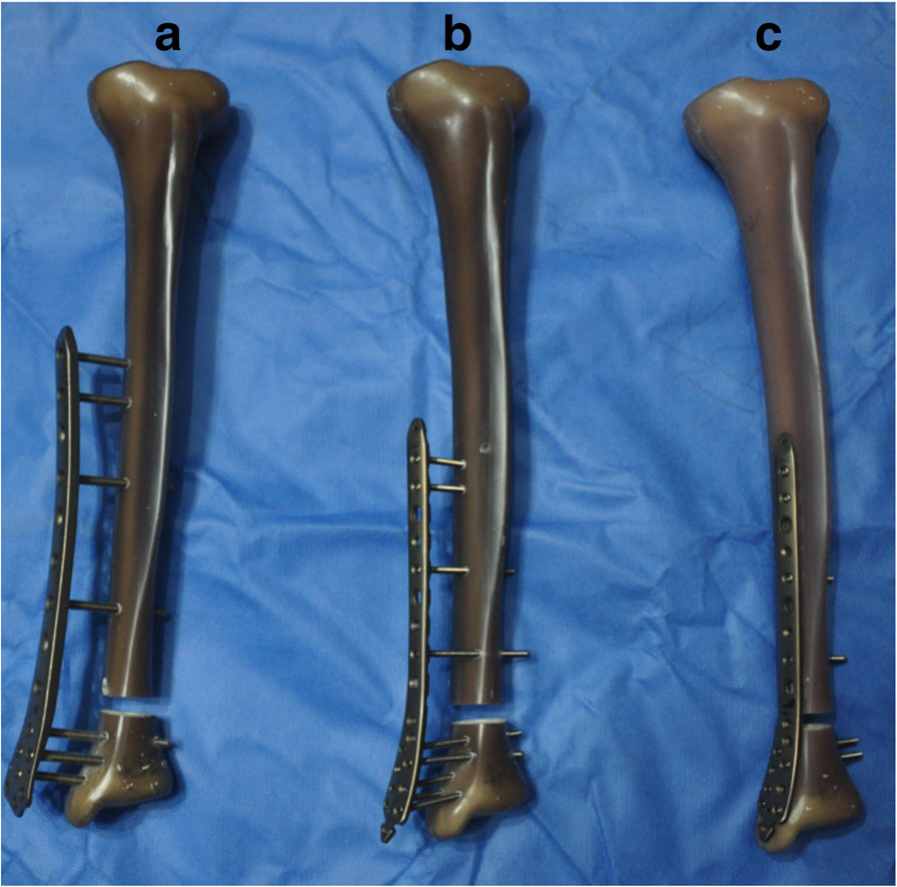
Examples of different models of specimen. The tibia in the external fixation (EF)-femur model (**a**) was plated on the medial aspect using an ure titanium 5-mm 9 + 7-hole LCP (medial distal femur plate, Kanghui), with a distance of 30-mm between the surface of the tibia to the lower surface of the plate. Standard, pure titanium 5-mm cortical screws were used in proximal slot nos. 1, 2, 4, and 7, as well as in distal slot nos. 10, 11, 12, and 13. The tibia in the EF-tibia model (**b**) was was plated on the medial aspect using a pure titanium 3.5-mm 9 + 10-hole LCP, with a distance of 30-mm between the surface of the tibia to the lower surface of the plate. Standard, pure titanium 3.5-mm cortical screws were used in proximal slot nos. 1, 2, 5, and 8, as well as in distal slot nos. 12, 13, 16, 17, and 18. The tibia in the LCP model (**c**) was plated on the medial aspect using a standard, pure titanium 3.5-mm 9 + 10-hole LCP, with standard, pure titanium 3.5-mm cortical screws used in proximal slot nos. 1, 2, 5, and 8, as well as in distal slot nos. 12, 13, 16, 17, and 18

Detailed procedures for each group were as follows:

In the EF-femur group (12 tibia in total), each tibia was plated on the medial aspect using an ure titanium 5-mm 9 + 7-hole LCP (medial distal femur plate, Kanghui), with a distance of 30-mm between the surface of the tibia to the lower surface of the plate. Standard, pure titanium 5-mm cortical screws were used in proximal slot nos. 1, 2, 4, and 7, and in distal slot nos. 10, 11, 12, and 13 (Fig. 1a). In the EF-tibia group (12 tibia in total), each tibia was plated on the medial aspect using a pure titanium 3.5-mm 9 + 10-hole LCP (medial distal tibia plate, Kanghui), with a distance of 30-mm between the surface of the tibia to the lower surface of the plate. Standard, pure titanium 3.5-mm cortical screws were used in proximal slot nos. 1, 2, 5, and 8, and in distal slot nos. 12, 13, 16, 17, and 18 (Fig. 1b). In the LCP group (12 tibia in total), each tibia was plated on the medial aspect using a standard, pure titanium 3.5-mm 9 + 10-hole LCP (medial distal tibia plate, Kanghui, Changzhou, China). Standard, pure titanium 3.5-mm cortical screws were used in proximal slot nos. 1, 2, 5, and 8, and in distal slot nos. 12, 13, 16, 17, and 18 (Fig. 1c).

### Biomechanical tests

For each tibia specimen in each group (LCP, EF-tibia, and EF-femur groups), axial compression stiffness test and torsion rigid test were performed to evaluate their stiffness of compression and torsion, with the mechanical tests and loading conditions following Yenna et al.’s study [15].

In the axial compression stiffness test, the tibia plateau was compressed using a custom-machined aluminum plate (Shimadzu, AG-10 kN IS, Kyoto, Japan). A steel bearing was placed under the centrally drilled hole of the tibia plafond, and the applied load was increasing until reaching the yield point for the LCP group and until closing the fracture gap for the EF-tibia and EF-femur groups.

Samples were loaded with a rate of 0.1 mm/second, and measured with a frequency of 100 Hz, with the load and displacement data recorded continuously.

In the torsion rigid tests, the tibia sample was placed horizontally using two custom-made jigs (Reger, RNJ-100, Shenzhen, China). The testing frame rotated the tibia across its central axis, and the applied load was increasing until reaching the yield point for the samples in the LCP, EF-femur, and EF-tibia groups. Samples were loaded with a rate of 0.5°/second, and were measured with a frequency of 100 Hz, with the load and displacement data recorded continuously.

### Statistical analysis

With the load and displacement data collected in the compression stiffness and torsion rigid tests, a load-displacement curve was computed for each sample in each group. Then the grand averaged load-displacement curve was obtained for LCP, EF-femur, and EF-tibia groups by averaging across samples in the corresponding groups.

Stiffness was identified as the slope of the linear portion of the curve using Excel 2007 (Microsoft, Seattle, WA, USA). Mean values and standard deviations of axial compression stiffness and torsional rigidity across samples in each group were calculated. Then one-way analysis of variance (ANOVA) with factor of ‘group’ (LCP, EF-femur, EF-tibia) was performed to evaluate the differences of axial and torsional stiffness of samples across groups using SPSS, version 17.0 (SPSS Inc., Chicago, IL, USA).

When the main effect was significant (*p* < 0.05), post hoc pairwise comparisons were used. Sample size in each group was sufficient to support the statistical analysis because there were few individual differences among samples due to the use of composite tibiae and the same measurement equipments.

## Results

**Fig. 2:**
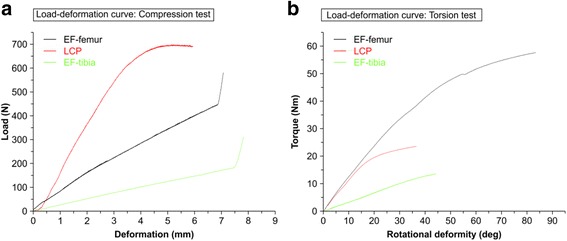
Load deformation curves in different models. The group-level load deformation curves of samples in EF-femur, LCP, and EF-tibia models were obtained in the axial compression and torsion rigid tests. The deformation curves were plotted in *black* line for EF-femur model, in *red* line for LCP model, and in *green* line for EF-tibia model. Panel **a**: x-axis, deformation (mm); y-axis, load (N). Panel **b**: x-axis, rotational deformity (deg); y-axis, toque (Nm)

As revealed by the group-level load-deformation curves in the axial compression stiffness tests (left panel of Fig. 2), the samples in the LCP group reached the yield point at the load of about 700 N, while those in the EF-femur and EF-tibia groups did not reach the yield point. Before the samples in the EF-femur and EF-tibia groups reached the yield point, the fracture gap was closed and stiffness increased rapidly. With the same amount of load applied to the samples, the samples in LCP groups showed minimal deformation compared with those in EF-femur and EF-tibia groups, indicating relatively high axis compression stiffness in LCP group. In addition, samples in EF-femur group showed higher compression stiffness than those in EF-tibial group. When the fracture segment bore was partially weighted (about 20 kg), the axial interfragmentary movements of samples in LCP, EF-femur, and EF-tibia groups were 1.1 mm, 2.3 mm, and 7.8 mm, respectively. The group-level torque-rotational deformity curves obtained in the torsional rigidity tests were shown in the right panel of Fig. 2. With the applied torque in the range of 0–20 Nm, the rotational deformity of samples in LCP and EF-femur groups were similar, both displaying less rotational deformity than those in the EF-tibia group.

**Fig. 3:**
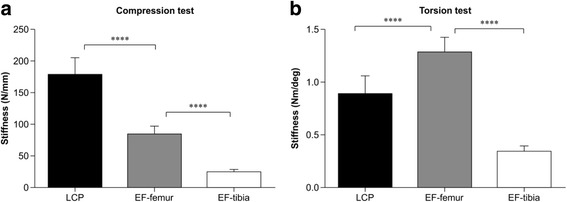
Comparison of compression stiffness and torsional rigidity in different models. Values of compression stiffness and torsional rigidity are displayed in *black*, *grey*, and *white* bars for LCP, EF-femur, and EF-tibia models respectively. Asterisk **** indicates a significant difference (*p* < 0.0001) between models

The axial compression stiffness across samples in each experimental group (left panel of Fig. 3) was summarized as follow: 177.9 ± 20.31 N/mm, 84.38 ± 14.37 N/mm, 25.04 ± 2.19 N/mm, for LCP, EF-femur, and Ef-tibia groups respectively, and the torsional rigidity across samples in each experimental group (right panel of Fig. 3) was summarized as follow: 0.89 ± 0.17 Nm/deg, 1.29 ± 0.14 Nm/deg, 0.34 ± 0.05 Nm/deg, for LCP, EF-femur, and Ef-tibia groups respectively. The mean LCP compression stiffness in EF-tibia and EF-femur groups were 14.07 and 47.43% of that in LCP group, respectively. The mean LCP torsional rigidity of in EF-femur and EF-tibia groups were 144.66% and 38.25% of that in LCP group, respectively. As revealed by 3-level (LCP, EF-femur, and Ef-tibia groups) one-way ANOVA, both compression and torsional stiffness were significantly different across groups (*p* < 0.05 for both comparisons). Post hoc tests revealed that (1) the samples in LCP group showed highest compression stiffness, compared with those in Ef-femur group (*p* < 0.001) and those in EF-tibia group (*p* < 0.001); (2) the samples in EF-femur group showed the highest torsional rigidity, compared with those in the EF-tibia group (*p* < 0.001) and those in the LCP group (*p* < 0.001).

## Discussion

The comparison of the stiffness of samples in the LCP, EF-femur, and EF-tibia groups revealed that the distance between the bone and the plate significantly reduced the rigidity of LCP. It has been shown that the main factors affecting the stiffness of LCP include working length, number of screws, distance from the plate to the bone, and length of the plates [16]; while construct stiffness is greatly affected by the fracture gap [14]. The differences of samples in LCP group and EF-tibia group were the distance between the bone and the plate, with other factors keeping consistent. The results of the comparison are consistent with previous report that the stiffness decreases with the increase of the bone-plate distance [12], and the distance used in this study, i.e., 30 mm, is the upper bound to keep external platefixation stable in a distal tibia fracture [13]. The bone-plate distances in EF-tibia and EF-femur groups were the same; while the medial distal femur plate had different diameters of the screws and dimensions of the plate from the medial distal tibial plate. The comparison between EF-tibia group and EF-femur group showed that increased diameters of the screws as well as the dimensions of the plate significantly enhanced torsional rigidity but contributed little to compression stiffness. The increased core diameters of the screws could significantly enhance the torsional strength at a rate proportional to the radius to the fourth power [17], indicating that a 5-mm screw is four times stiffer than a 3.5-mm screw.

Hoenig et al. ever reported that the mean compression stiffness is 72.5 N/mm for a standard plate, 122 N/mm for an LCP, and 179 N/mm for an IM [18], and Yang et al. ever showed that the Ilizarov fixator’s stiffness ranged within 73–79 N/mm [19]. Though the characteristics of the fixator construction and the loading modes were usually different in these studies, their results on axial compression were still helpful in assessing the rigidity of LCP as an external fixator. According to these results, it was found that the distal femur LCP as an external fixator had approximately the same stiffness as the standard plate or the Ilizarov fixator.

A flexible fixator construct can easily lead to excessive interfragmentary movements, which would hinder fracture healing and lead to delayed union or nonunion [20]. The comparison of axial compression stiffness of the samples in LCP, EF-femur, and EF-tibia groups showed that the samples’ constructs in EF-tibia group were too flexible. Secondary fracture healing requires stiffness to be within an optimal range [21], which is still unclear due to the fact that the optimal mechanical environment will change during the healing process of a given fracture. Thus, it is hard to say that constructs in the EF-tibia or EF-femur group were unsuitable for treating distal tibial fractures; yet, EF-tibia and EF-femur groups were associated with the potential risk of delayed union and nonunion based on the obtained results in the present study.

To the best of our knowledge, the present study is one of few reports quantitatively evaluating the biomechanical characteristics of LCP as an external fixator for treating distal tibial fractures. Ma et al. and Zhang et al. investigated the finite element analysis model, but didn’t make a comparison of stiffness between external plating and conventional LCP [9, 13]. Kanchanomai et al. and Ahmad et al. reported a tibial shaft fracture model, instead of a distal tibial fracture model [12, 14]. Though the LCP technique has shown a high union rate in clinical practice, its popularity and acceptance were still blocked by its construct stiffness. Samples in EF-femur group had exhibited more rigid constructs than those in the EF-tibia group, indicating that the advantage of EF-femur configuration in distal tibial fracture treatment.

Indeed, several limitations exist in the present biomechanical study, including (1) samples used in the experiments were composite tibia rather than real bone. Even though composite tibia has been validated as a suitable substitute for cadaver specimens [22], it cannot provide a real in-vivo environment where the fibula and complex muscular interactions can help enhance the stability of the tibia; (2) the results of the biomechanical tests cannot be directly extrapolated to the clinical setting; (3) the loading design in the biomechanical tests did not account for the loading’s multifaceted manner that occurs in humans, i.e., axial load is the most clinically significant load experienced by patients during rehabilitation compared with transverse and torsional loads.

## Conclusions

The present study comprehensively evaluated the biomechanical characteristics of specimens in LCP group (i.e., with a medial distal tibial LCP), EF-tibia group (i.e., with medial distal tibial LCP with 30-mm plate-bone distances), and EF-femur group (i.e., with medial distal femur LCP with 30-mm plate-bone distances). The flexible construct compression stiffness of both distal femur LCP and distal tibial LCP, as revealed in the test, suggested that potential risks of delayed union or nonunion existed in both techniques; distal femur LCP would be a better one due to its higher axial compression stiffness and torsional rigidity. Given all advantages and risks, distal femur LCP was recommended as an external fixator in treating distal tibial fractures, but should be used cautiously.

## Abbreviations

ANOVA: Analysis of variance
EF: External fixation
IM: Intramedullary nailing
LCP: Locking compress plates
MIPPO: Minimally invasive percutaneous plate osteosynthesis
